# Supercritical Carbon Dioxide Extraction of Carotenoids from Pumpkin (*Cucurbita* spp.): A Review

**DOI:** 10.3390/ijms15046725

**Published:** 2014-04-21

**Authors:** Miriana Durante, Marcello Salvatore Lenucci, Giovanni Mita

**Affiliations:** 1 Istituto di Scienze delle Produzioni Alimentari–Consiglio Nazionale delle Ricerche (ISPA-CNR), Via Prov. le Lecce-Monteroni, Lecce 73100, Italy; E-Mail: giovanni.mita@ispa.cnr.it; 2 Dipartimento di Scienze e Tecnologie Biologiche ed Ambientali (Di.S.Te.B.A.), Università del Salento, Via Prov. le Lecce-Monteroni, Lecce 73100, Italy; E-Mail: marcello.lenucci@unisalento.it

**Keywords:** carotenoids, conventional solvent extraction, co-matrix, co-solvent, *Cucurbita* spp. modifier, supercritical carbon dioxide

## Abstract

Carotenoids are well known for their nutritional properties and health promoting effects representing attractive ingredients to develop innovative functional foods, nutraceutical and pharmaceutical preparations. Pumpkin (*Cucurbita* spp.) flesh has an intense yellow/orange color owing to the high level of carotenoids, mainly α-carotene, β-carotene, β-cryptoxanthin, lutein and zeaxanthin. There is considerable interest in extracting carotenoids and other bioactives from pumpkin flesh. Extraction procedures able to preserve nutritional and pharmacological properties of carotenoids are essential. Conventional extraction methods, such as organic solvent extraction (CSE), have been used to extract carotenoids from plant material for a long time. In recent years, supercritical carbon dioxide (SC-CO_2_) extraction has received a great deal of attention because it is a green technology suitable for the extraction of lipophylic molecules and is able to give extracts of high quality and totally free from potentially toxic chemical solvents. Here, we review the results obtained so far on SC-CO_2_ extraction efficiency and quali-quantitative composition of carotenoids from pumpkin flesh. In particular, we consider the effects of (1) dehydration pre-treatments; (2) extraction parameters (temperature and pressure); the use of water, ethanol and olive oil singularly or in combination as entrainers or pumpkin seeds as co-matrix.

## 1. Introduction

Carotenoids are largely used in food preparation to make product color appealing to consumers. They represent a group of bioactive compounds responsible for the yellow-red pigmentation of many plant organs and plant derived raw and processed foods, known to improve food healthiness and stability because of their pro-vitamin A activity, antioxidant power and boost of cell-mediated and humoral immune response [1,2].

Of the more than 700 natural carotenoids identified up to now, about 50 could potentially be absorbed, transported, delivered to tissues, metabolized and, eventually, used by the human body. The number is reduced to six (α-carotene, β-carotene, lycopene, lutein, zeaxanthin and β-cryptoxanthin) considering only those effectively detected in blood plasma and tissues of people from different countries and associated with some health benefits [3,4].

The Mediterranean diet offers a high amount and a wide diversity of carotenoids due to the regular intake of fruit and vegetables. Tomato, green leafy vegetables (spinach, broccoli, endive, lettuce, *etc*.) cereals and red-pepper are the main sources of lycopene, lutein, zeaxanthin and β-cryptoxanthin, respectively. While α-carotene and β-carotene are taken up mainly by the consumption of carrot, sweet potato and pumpkin [4].

Pumpkin is thought to be native of the South America; it belongs to the genus *Cucurbita* of the *Cucurbitaceae* family. *C. moschata*, *C. maxima* and *C. pepo*, are three of the most cultivated species world-wide [5]. They are frequently consumed cooked as a main course or side dish, and used, as an ingredient, in pies, soups, stews, and bakery preparations [6,7]. Pumpkin is also popular in traditional medicine for several ailments (antidiabetic, antihypertensive, antitumor, immunomodulation, antibacterial, antihypercholesterolemia, intestinal antiparasitia, antiinflammation, antalgic) [8]. The presence of many biologically active components including high levels of α- and β-carotene, β-cryptoxanthin, lutein and zeaxanthin, polysaccharides, phytosterols, unsaturated fatty acids, proteins and peptides makes pumpkin extremely attractive for the phytochemical manufacturing industry [9,10].

Different technological approaches have been developed to extract bioactive molecules from pumpkin, especially oil and proteins from seeds [11]. Conventional solvent extraction (CSE) and supercritical CO_2_ (SC-CO_2_) extraction were reported to be effective methods to produce pumpkin seed oils, with the latter being particularly convenient to obtain solvent-free extracts with unaltered pharmacological activities [12]. In the last five years, several papers have focused on carotenoid extraction from pumpkin flesh, expanding the potential use of the fruit mesocarp previously proposed exclusively as a source of cell wall polysaccharides such as pectins [13,14]. These studies investigated the effects of extraction parameters and plant material pretreatments (dehydration, granulometry, *etc*.) on carotenoid yield and composition [15–18].

Large scale carotenoid extraction from ripe pumpkin fruits could give a product useful as ingredient for functional food, cosmeceutical and pharmaceutical preparations. Traditionally, CSE use *n*-hexane, propanol, methanol, tetrahydrofuran or ethyl acetate to extract carotenoids [19,20]. This method usually requires long extraction time, large amounts of organic solvent and high temperatures which could lead to extensive degradation of thermo-sensible molecules such as carotenoids and often leave trace amounts of potentially toxic solvent in the extract [21–23].

SC-CO_2_ extraction technology has been widely used as an alternative to CSE for the extraction of natural products because it gives extracts totally free of organic solvents [24,25]. As a solvent, CO_2_ is non-toxic, non-flammable, readily available, low cost and exhibits high selectivity as a result of low viscosity, high diffusivity and liquid-like density [26,27]. In addition, CO_2_ has a low critical temperature and pressure (31 °C and 74 bar, respectively) which makes it the ideal solvent for the extraction of thermo-sensitive molecules such as carotenoids [28]. Many studies have been conducted to investigate the applicability of SC-CO_2_ for the extraction of bioactive compounds [29,30] and several authors focused their research on the carotenoid extraction from a wide range of plant material such as leaf protein concentrates [31], paprika [32], sweet potato [33], tomato [34], watermelon [35] and wheat bran [25,36].

In a recent paper, the chemical, nutritional and technological aspects of pumpkin as a source of bioactive molecules, as well as the health-benefits associated with its consumption have been reviewed [37]. Here, we summarize the most recent findings related to carotenoid extraction from pumpkin flesh with an emphasis on SC-CO_2_ extraction and related issues such as pumpkin flesh dehydration, matrix granulometry, use of entrainers or co-matrices.

## 2. (SC-CO_2_) Extraction of Carotenoids from Pumpkin

### 2.1. Effect of Pre-Treatment

Drying is one of the most ancient methods to preserve or process food, including fruits and vegetables. Dried vegetable powders are often incorporated in food products (soups, noodles, bread, cakes and pasta) to improve their flavor and nutritional value [38].

Recently, dehydration gained great attention especially in industrial processes that use SC-CO_2_ to extract phytochemicals. The high water content of many plant tissues and organs is detrimental to the efficient extraction of lipophilic nutraceuticals with SC-CO_2_. Being polar and scarcely soluble in SC-CO_2_, water hinders the fluid flow through the plant tissues reducing the contact surface between solutes and solvent and increasing the diffusion resistance of solutes [18,24,39]. It also leads to problems such as restrictor or valve clogging due to ice formation and difficulties in removal of water from the extracts [40,41]. Moisture reduction is, hence, a prerequisite to improve the yield and quality of SC-CO_2_ extracted liposoluble molecules from plant tissues. It is known that moisture content, within certain percentages, influence SC-CO_2_ extraction of lipophilic molecules [42].

Hot air- [43], oven- [44–46], freeze- [44,47,48], convective- [44,49–51], microwave- [52,53], and osmotic- [54–56] drying methods, as well as combined treatments such as microwave-air [53] and vacuum-microwave [44], have been assessed for pumpkin flesh dehydration, and their effects on drying kinetics, chemical properties (moisture, ash, total and reducing sugars, fibers, proteins, lipids, and isoprenoids), texture and color of the final products have been investigated [7,16,47].

Due to its affordability, oven-drying is the most common dehydration process, but it often leads to the degradation of thermolabile compounds and/or oxidizable substrates such as carotenoids, tocochromanols and lipids. It is known that high temperature may alter the chemical composition, the color, the nutritional value and the organoleptic quality of the dried product. Color changes have been related to carotenoids degradation during pumpkin heat processing [43,57]. Nevertheless, only a few papers report data on the carotenoid composition of dehydrated pumpkin flesh (Table 1). Using conventional solvent extraction techniques, Wang *et al*. [43] found that hot-air treatment (70 °C) of pumpkin flesh slices (*C. maxima*), with 9.3% residual moisture, determined a strong reduction (65%) of total carotenoid content with respect to raw pumpkin (67.6 mg/100 g). In order to overcome these limitations, oven-drying is generally carried out under vacuum, at moderate temperature (40–60 °C) [44,45] or replaced by freeze-drying [45,48] which preserves, almost unaltered, the physico-chemical characteristics and quality of the raw plant material [45]. However, the high cost of this process is a major drawback of this method, justified only for extracts with high commercial value [58]. Dirim and Çalıskan [38] obtained a freeze-dried pumpkin matrix (*C. moschata*), with a residual moisture of 3.9% and total carotenoid content [0.7 mg/100 g dry matter (d.m.)] approximately 26% lower than that of the fresh material (1.1 mg/100 g d.m.). An approximately 6% reduction of total carotenoids, with respect to raw pumpkin, was reported by Wang *et al*. [43] in freeze-dried pumpkin slices with a residual moisture of 10.9%. Nawirska *et al*. [45] evaluated the total carotenoid content of dried slices of 12 pumpkin (*C. maxima* and *C. pepo*) cultivars, the highest average total carotenoid content was obtained from freeze-dried samples (160 mg/100 g d.m.), followed by vacuum microwave (130 mg/100 g d.m.) and by convective-drying (5 mg/100 g d.m.). More specifically, Durante *et al*. [16] compared the effects of vacuum oven- and freeze-drying methods on carotenoids concentration in a dehydrated pumpkin matrix. Oven- and freeze-dried pumpkin had a residual moisture of approximately 8% and 12% *w*/*w*, respectively (Table 1). No statistically significant difference in total carotenoid yields was observed between oven- or freeze-dried matrices (81.3 *vs.* 73.7 mg/100 g d.m., respectively). Wang *et al*. [43] proposed a dehydration method based on treatment of pumpkin slices with a red algae extract (RAE) at 40% (*w*/*w*), a promising new dehydrating agent. They found that RAE-treated samples had a residual moisture of 7.8% and a total carotenoid content approximately 15% lower than that measured in raw pumpkin and considerably higher than in hot air-dried pumpkin slices.

In studies conducted on the SC-CO_2_ extraction of carotenoids from pumpkin (Table 2), Shi *et al*. [17] used freeze-dried *C. moschata* flesh matrix, containing a 10% residual moisture, to feed the extractor, getting a maximum carotenoid yield of about 10.9 mg/100 g d.m. The same authors, using freeze-dried matrix from *C. maxima* obtained a carotenoid amount of 132.2 mg/100 g. Conversely, Durante *et al*. [16] reported that total carotenoid yield obtained by SC-CO_2_ from oven-dried pumpkin flesh (*C. moschata*) was about 8.5 fold higher than from freeze-dried sample (49.2 *vs.* 5.8 mg/100 g d.m., respectively). The higher carotenoid yield, obtained from oven-dried matrix, was hypothesized to be due to the prolonged heat treatment on plant tissue and cell structure such as cell membranes and chloroplasts that might cause the disaggregation and cell wall loosening, and a consequent increase in the permeability of the matrix to the fluid, as also suggested by Gutiérrez *et al*. [59] and Snyder *et al*. [60]. Furthermore, it has been reported that freeze-drying may strengthen tissue aggregation leading to a decrease in solute-solvent contact surface which negatively affects extraction yields [61,62].

The amount of carotenoids extracted by SC-CO_2_ is also affected by the particle size of the dried sample. Generally, the smaller the particle size, the higher the amount of extracted carotenoids [63]. This is due to the higher surface to volume ratio of smaller particles and to a reduction of the path length that analytes must diffuse through to reach the bulk phase [64,65]. On the other hand, the use of very small particles could be detrimental. They can excessively compact the extraction bed, increasing the internal mass transfer resistance and causing channeling effects [66]. To our knowledge, there are no substantial data about the effect of particle size on carotenoid extraction from dried pumpkin flesh. Durante *et al*. [16] carried out SC-CO_2_ extraction of carotenoids from pumpkin on samples with a particle size of 250 μm, corresponding to 70 mesh, while Shi *et al*. [17,18] used samples milled to a size of 1 mm, corresponding to 18 mesh, however, because of the extensive differences in matrix pre-treatment and extraction parameters the results are hardly comparable.

### 2.2. Effects of Temperature and Pressure

Supercritical fluid extraction solubility and selectivity depend greatly on the equilibrium between fluid density and solute vapor pressure which are affected by temperature and pressure. Generally, at constant pressure, the increase of temperature reduces the density of SC-CO_2_ thus reducing its solvating power, but it enhances the vapor pressure of extractable compounds, consequently increasing analyte solubility and extraction yield [66]. At constant temperature, the fluid density increases with increasing pressure and, accordingly, increases the solubility of the analyte [67].

Table 3 summarizes the findings regarding the effect of temperature and pressure on carotenoids composition extracted by SC-CO_2_ from pumpkin flesh.

Shi *et al*. [17] utilized different temperatures from 40 to 70 °C and pressures from 25 to 35 MPa in SC-CO_2_ extraction of carotenoids from pumpkin (Table 3). They found that carotenoid yield increased with increasing pressure and temperature. The highest extraction yield (6.1 mg/100 g matrix d.m.) was obtained at 70 °C and 35 MPa and highlighted the importance of the interaction of the two parameters in affecting SC-CO_2_ extraction of carotenoids from pumpkin matrices.

A particularly important effect of temperature regards the isomerization and degradation of extracted plant carotenoids. In plant tissues most carotenoids occur naturally as all-*E*-isomers which can be easily isomerized to *Z*-isomers when exposed to heat and light. It has been reported that the increase in extraction temperature over 80 °C is accompanied by carotenoids isomerization and degradation [24,68]. Shi *et al*. [17] observed that in SC-CO_2_ pumpkin extracts, when the extraction temperature was raised from 40 to 70 °C, the (9 + 13)-*Z*-β-carotene isomers increased significantly from 10.3% to 12.7% at 25 MPa and from 8.0%–12.5% at 35 MPa (Table 3). Shi *et al*. [18] found that when the extraction temperature was increased from 50 to 80 °C at a constant pressure of 25 MPa, carotenoid yield decreased from 132.2 to 44.6 mg/100 g d.m. and the ratios of *Z*-β-carotene/total carotenoids increased from 13.7% to 24.5%. Durante *et al*. [16] reported that, at 35 MPa and 60 °C, SC-CO_2_ extraction of oven-dried pumpkin matrix gave an extract in which *Z*-isomers (represented only by 13-*Z*-β-carotene) contributed to approximately 9.3% to the total carotenoid amount.

Although SC-CO_2_ flow-rate is an important parameter affecting SC-CO_2_ carotenoid extraction, no literature data have been available for pumpkin so far.

### 2.3. Effects of Entrainers and Co-Matrices

Pure SC-CO_2_ has good solvent properties for extraction of non-polar and moderately polar compounds which can be suitably tailored by varying temperature and pressure. To extend SC-CO_2_ solvating power, improve its affinity for poorly soluble solutes and, ultimately, increase the extraction yield, small volumes of entrainers (modifier or co-solvents) such as water, methanol, ethanol, hexane, *etc*., are often added. The chemical features of modifiers and their concentration within the supercritical fluid are the key parameters affecting solubility of a target solute, nevertheless the physical structure of the solid matrix (granulometry, hydration, packing density), as well as its chemical composition, must be taken into account to obtain good extraction yields [69]. Entrainers could be constantly added to the CO_2_ mobile phase upstream of extraction vessel (co-solvent) or, alternatively, mixed with the matrix powder into the extractor vessel (modifier). In the latter case, modifiers, depending on their solubility in SC-CO_2_, can be quickly transported out of the extractor by the supercritical fluid with the consequent loss of their positive effects. In order to overcome this limitation, co-extraction procedures consisting in blending the matrix with appropriate co-matrices naturally providing the entrainer (usually a vegetable oil) have been successfully developed [70–73].

Table 3 reports the data published so far on the effect of entrainers or co-matrix addition on total yield and composition of carotenoid extracts obtained from pumpkin by SC-CO_2_ extraction.

Investigating the effects of water, ethanol or olive oil added as modifiers for SC-CO_2_ extraction of carotenoids from pumpkin flesh, Shi *et al*. [17,18] found that most of them, used either alone or in combinations, had significant effects on both yield improvement and composition of carotenoid extracts (Table 3). Temperature strongly affected carotenoid yields since, as reported by Shi *et al.* [17], there is a clear improvement at 70 °C with respect to 40 °C. Furthermore, a reduction in carotenoid yield was observed at 80 °C, confirming that this could be considered a limit temperature inducing thermal denaturation of extracts [18].

Regardless of temperature the effect of single modifier on the extraction yield followed the order: olive oil > ethanol > water. While the highest yield was obtained at 50 °C using 10% ethanol + 10% oil as modifier.

Relatively low water contents (5% and 10%) were not effective in increasing total carotenoid yield at extraction temperature of 50 °C, however 15% water improved the carotenoid extraction of 1.35-fold. As previously stated (see Section 2.1), excess of water is generally detrimental for SC-CO_2_ extraction of lipophilic compounds. Water polarity reduces the solvating power of SC-CO_2_ towards relatively non-polar carotenoids; besides it often causes mechanical problems due to ice formation [74]. Nevertheless, its positive effect in matrix swelling may facilitate CO_2_ diffusion through the sample and enhance carotenoid extraction. Swelling behavior is mainly controlled by temperature, this could explain the differences observed at 80 °C operating temperature. In this case, the tendency was the opposite, the higher the water content the lower the yield improvement [18]. The presence of polar co-solvents such as ethanol or methanol increases the polarity of the medium and simultaneously increases the overall recovery yield of polar molecules. Ethanol has been broadly used as a co-solvent in extraction of bioactive compounds due to its low toxicity compared to other solvents [75–77]. Shi *et al*. [18] observed that the effect of ethanol was greater than that of water on carotenoid yield at both temperatures (50, 80 °C) assayed, because the addition of ethanol increased the bulk density of SC-CO_2_ due to the high density of the co-solvent and clustering of SC-CO_2_ molecules around the co-solvent [78]. These results are in agreement with the previous findings of the same research group [17] who reported that the use of 10% ethanol as modifier doubled carotenoid extraction yield from pumpkin flesh.

In some reports, vegetable oils were used as modifier. Sun and Temelli [79] found that the use of canola oil increased SC-CO_2_ extraction yield of carotenoids from carrots. This is possibly due to the interaction of canola oil with the matrix, thus facilitating the solubilization of carotenoids, or swelling of the matrix. Shi *et al*. [18] reported that olive oil enhanced the extraction yield by increasing the solubility of α-carotene, β-carotene, (9 + 13-*Z*)-β-carotene, lutein and lutein esters in the modified supercritical fluid. When a mixture of two modifiers was used, carotenoid yield was even higher than that obtained using equivalent amounts of single modifiers. Such enhancement increased with the increasing amount of both ethanol and olive oil (either at 50 or 80 °C). The results obtained at 50 °C showed that the combination of modifiers on the total carotenoids yield followed the order: water/oil > ethanol/oil > water/ethanol, while at 80 °C the combination of modifier on the total carotenoid yield followed the order: oil/ethanol > water/oil > water/ethanol. Olive oil (-COOR) has H-bonding ability, which could link to ethanol and contribute to the interaction between modified SC-CO_2_ and the analyte improving the solubility of carotenoids. Furthermore, the addition of modifiers increased the antioxidant activities of the extracts probably due to the percentage of polar and non-polar compounds extracted by SC-CO_2_. Several studies indicate that β-carotene and α-tocopherol act synergistically and α-tocopherol protects β-carotene from oxidation [80–82].

Shi *et al*. [18] described that the modifier and the operating temperature notably influenced the ratio of *Z*-β-carotene/total carotenoids extracted by SC-CO_2_. The antioxidant activity did not show a linear correlation with the total yield of carotenoids, it could be related to the proportion of all-*E* form and *Z-*isomers present in extracts, as well as the ratio of different type *Z*-isomers, such as 5-*Z*-β-carotene, 9-*Z*-β-carotene and 13-*Z*-β-carotene, because some reports indicated that *Z*-forms of carotenoids had higher antioxidant activity compared to that of all-*E*-β-carotene [83–85].

Durante *et al*. [16] used milled pumpkin seeds as co-matrix and demonstrated that pumpkin seeds contributed to enhance the carotenoid extraction efficiency possibly by increasing the solubility of carotenoids in the CO_2_ and/or the fluid flow rate of carotenoids through the matrix and pipelines (Table 3). Moreover, the addition of pumpkin seeds to the pumpkin matrix led to an enrichment of the extract in Vitamin E and polyunsaturated fatty acids.

### 2.4. Comparison of Carotenoid Composition Extracted from Pumpkin by Organic Solvent and SC-CO_2_

Conventional solvent extraction (CSE) has been widely used to extract bioactive compounds from plants. Despite its numerous drawbacks, including long extraction time and consumption of large amounts of potentially toxic solvents, CSE is often used as benchmark for other extraction methods. CSE and SC-CO_2_ carotenoid extraction efficiency from pumpkin flesh and carotenoid profile of extracts have been recently compared [16–18]. Table 4 reports the composition of carotenoids extracts obtained from pumpkin by both CSE and SC-CO_2_.

Shi *et al*. [17] obtained the highest total carotenoids yield from ripe pumpkin (*C. moschata*) using ethanol/hexane (1:1 *v*/*v*), lower yields were obtained by SC-CO_2_ extraction at 70 °C and at 40 °C with or without the addition of ethanol, as modifier (Table 4). Total carotenoid amounts extracted from *C. moschata* were lower than those described by Durante *et al*. [16] possibly depending on several factors such as genotype, pedoclimatic conditions, variation in harvesting time, post harvesting storage, matrix preparation, *etc*. However, also in this study, the amount of carotenoids obtained after hexane extraction, from pumpkin matrix, was about 1.6-fold higher than those obtained after SC-CO_2_. The addition of milled pumpkin seeds to the matrix contributed to enhance carotenoid extraction by SC-CO_2_, in fact, in these experimental conditions the amount of carotenoids extracted by either hexane and SC-CO_2_ were not significantly different [16].

Shi *et al*. [17] described that lutein and lutein esters, lycopene and the total amount of 9-*Z* and 13-*Z*-β-carotene were higher inSC-CO_2_ extracts obtained at 70 °C (using etanol as modifier) than in CSE extracts and similar results were obtained by Shi *et al*. [18].

## 3. Conclusions

SC-CO_2_ extraction of carotenoids from pumpkin have gained great attention in the last few years. The studies here reviewed show that pumpkin SC-CO_2_ extracts are interesting, innovative, and high quality products rich with α- and β-carotene as well as other bioactive compounds. Optimization of experimental parameters, such as pressure, temperature and moisture of pumkin matrices, and the use of entrainers or oleaginous co-matrix addition, can substantially increase carotenoid extraction yield. Vacuum oven- and freeze-drying pre-treatments gave good results in the preparation of pumpkin matrices suitable for supercritical CO_2_-extraction. However, vacuum oven-dried matrices gave far better results in terms of carotenoid extraction yield than oven-dried matrices possibly due to tissue and cell structure disaggregation which may increase the permeability of the matrix to the fluid. The best operative conditions for pumpkin carotenoids extraction by SC-CO_2_ were found to be: 35 MPa, 50–70 °C, for pressure and temperature, respectively. The highest extraction yield was achieved by the combination of water (10%) and olive oil (10%) or ethanol (10%) and olive oil (10%), as entrainers, at 50 and 80 °C, respectively. Information on the influence of different seeds, as co-matrix, still require thorough studies.

Although the operation cost of SC-CO_2_ remains high and is still difficult to scale up the extraction process to an industrial level, these findings encourage further studies in order to scale up the process for possible industrial production of high quality bioactive ingredients from pumpkin for nutraceutical, cosmeceutical and pharmaceutical preparations. It is also desirable that comparative research trials were undertaken to assay any differences in the biological activity and action mechanisms of carotenoid containing SC-CO_2_ extracts from pumpkin compared to those obtained by conventional extraction techniques.

## Figures and Tables

**Table 1.** Carotenoid composition of dehydrated pumpkin flesh by conventional solvent extraction.

Reference	Extraction conditions				Total carotenoids (mg/100 g d.m.)

	Pumpkin species	Drying methods	Residual moisture (%, *w*/*w*)	Extraction methods	
Nawirska *et al.* [44]	C. *maxima*/ C. *pepo*	Vacuum microwave	-	Acetone 80% (*v*/*v*)	130
	C. *maxima*/ C. *pepo*	Convective	-	Acetone 80% (*v*/*v*)	5
	C. *maxima*/ C. *pepo*	Freeze-drying	-	Acetone 80% (*v*/*v*)	160

Wang *et al.* [43]	C. *maxima*	Hot-air	9.3	Acetone 100% (*v*/*v*)	67.6
	C. *maxima*	Freeze-drying	10.9	Acetone 100% (*v*/*v*)	63.7
	C. *maxima*	RAE (40%) *	7.8	Acetone 100% (*v*/*v*)	57.4

Dirim and Çalıskan [38]	C. *moschata*	Freeze-drying	3.9	Hexane/acetone/ethanol (50/25/25, *v*/*v*/*v*)	0.7

Durante *et al.* [16]	C. *moschata*	Vacuum oven	12	Hexane 100% (*v*/*v*)	81.3
	C. *moschata*	Freeze-drying	8	Hexane 100% (*v*/*v*)	73.7

* RAE, red algae extract.

**Table 2.** Effect of pre-treatment on carotenoid extracts from pumpkin by SC-CO_2_.

Reference	Extraction conditions				Total carotenoids (mg/100 g d.m.)

	Pumpkin species	Drying methods	Particle size (mesh)	Residual moisture (%, *w*/*w*)	
Shi *et al*. [17]	C. *moschata*	Freeze-drying	18	10	10.9
Shi *et al.* [18]	C. *maxima*	Freeze-drying	18	-	132.2
Durante *et al.* [16]	C. *moschata*	Vacuum oven	70	12	49.2
	C. *moschata*	Freeze-drying	70	8	5.8

**Table 3.** Effects of temperature, pressure, entrainers and co–matrix on carotenoids composition obtained by SC-CO_2_ from pumpkin.

Reference	Extraction conditions				Carotenoid composition (% of total)						Total carotenoids (mg/100 g matrix d.m.)
	
	Temperature (°C)	Pressure (MPa)	Entrainer	Co-matrix	Lutein and lutein esters	α-carotene	β-cryptoxanthin	β-carotene	(9 + 13-*Z*)-β-carotene	Lycopene	
Shi *et al.* [17]	40	35	-	-	41.7	14.6	-	34.4	7.3	2.0	1.6
	70	35	-	-	30.5	14.0	-	31.0	17.9	6.6	6.1
	40	25	10% E	-	33.4	15.3	-	35.0	10.3	5.8	2.9
	70	25	10% E	-	37.6	12.0	-	31.4	12.7	6.4	9.7
	70	35	10% E	-	38.2	11.4	-	32.8	12.5	5.2	10.9
Shi *et al.* [18]	50	25	-	-	28.8	14.1	-	43.3	13.7	-	132.2
	50	25	5%–15% E	-	25.8–25.9	14.9–15.6	-	45.5–44.9	13.8–13.6	-	161.9–217.7
	50	25	5%–15% W	-	16.2–25.4	17.4–15.8	-	47.6–45.1	17.1–13.7	-	118.8–179.1
	50	25	5%–15% O	-	19.3–28.1	16.9–17.5	-	43.5–42.9	10.5–11.5	-	245.0–254.1
	80	25	-	-	29.3	10.3	-	35.9	24.5	-	44.6
	80	25	5%–15% E	-	31.8–25.9	12.7–15.1	-	34.8–44.9	20.8–14.1	-	60.9–153.9
	80	25	5%–15% W	-	22.8–24.2	14.2–14.3	-	44.8–44.6	18.2–16.3	-	191.5–107.2
	80	25	5%–15% O	-	15.2–21.3	19.2–17.7	-	53.4–49.1	12.2–11.9	-	229.2–256.4
	50	25	10% W + 10% O	-	30.9	14.9	-	44.7	9.4	-	338.1
	50	25	10% O + 10% E	-	16.0	18.8	-	52.1	12.9	-	285.0
	50	25	10% W + 10% E	-	28.03	11.9	-	44.9	15.1	-	195.9
	80	25	10% W + 10% O	-	28.4	16.1	-	42.6	12.9	-	304.9
	80	25	10% W + 10% E	-	27.2	15.1	-	41.7	15.9	-	99.6
	80	25	10% E + 10% O	-	22.03	13.7	-	42.4	21.9	-	334.8
Durante *et al.* [16]	60	35	-	-	4.4	38.5	1.1	46.7	9.3	-	49.2
	60	35	-	Pumpkin seeds	5.0	33.3		0.7	55.5	5.5	- 79.2

**Table 4.** Comparison of carotenoids composition extracted from pumpkin by organic solvent and SC-CO_2._

Reference	Extraction conditions					Carotenoid composition (% of total)						Total carotenoids (mg/100 g matrix d.m.)
	
	Temperature (°C)	Pressure (MPa)	Extraction methods	Entrainer	Co-matrix	Lutein and lutein esters	α-carotene	β-cryptoxanthin	β-carotene	(9 + 13-*Z*)-β-carotene	Lycopene	
Shi *et al.* [17]	-	-	Ethanol/hexane (1/1, *v*/*v*)	-	-	35.4	19.7	-	41.4	3.5	0.1	14.8
Durante *et al.* [16]	69	0.1	Hexane	-	-	3.2	30.9	0.4	50.3	15.2	-	81.3
	69	0.1	Hexane	-	Pumpkin seeds	2.6	35.1	3.4	58.8	nd	-	84.0
Shi *et al.* [18]	-	-	Ethanol/hexane (4/3, *v*/*v*)	-	-	27.8	20.1	-	43.1	8.9	-	1286.4

nd, not detectable.
